# The Prognostic and Clinicopathological Roles of PD-L1 Expression in Colorectal Cancer: A Systematic Review and Meta-Analysis

**DOI:** 10.3389/fphar.2019.00139

**Published:** 2019-02-28

**Authors:** Yan Li, Meizhi He, Yaoyao Zhou, Chen Yang, Shuyi Wei, Xiaohui Bian, Odong Christopher, Lang Xie

**Affiliations:** ^1^Department of General Surgery, Zhujiang Hospital of Southern Medical University, Guangzhou, China; ^2^The Second School of Clinical Medicine, Southern Medical University, Guangzhou, China; ^3^The First School of Clinical Medicine, Southern Medical University, Guangzhou, China

**Keywords:** colorectal cancer, PD-L1/ PD-1, prognostic, clinicopathological, meta-analysis

## Abstract

**Background:** Studies evaluating the prognostic significance of programmed death-ligand 1 (PD-L1) expression in colorectal cancer (CRC) are limited and remain controversial. This meta-analysis was conducted in order to evaluate the clinicopathological and prognostic significance of PD-L1 expression in CRC patients.

**Methods:** A comprehensive search was performed against the Medline/PubMed, Embase, Cochrane Library, Web of Science (WoS) and Scopus databases. Data were extracted with name of the first author, year of publication, country of origin, tumor type, number of cases, staining method, cut-off values, PD-L1 positive expression, clinicopathological parameters, outcome, and quality assessment score, and statistical analysis was conducted using Review Manager Version 5.3 (Revman the Cochrane Collaboration; Oxford, England) and STATA version 14 (Stata Corporation; College Station, TX, USA).

**Results:** Ten studies were included in this meta-analysis, in which the pooled hazard ratio (HR) showed that PD-L1 expression in tumor cells was significantly associated with a poor overall survival (HR = 1.50, 95% CI 1.05–2.13, *P* = 0.03). The pooled HR for disease-free survival (DFS) indicated that PD-L1 expression was significantly associated with shorter DFS (HR = 2.57, 95% CI 1.40–4.75, *P* = 0.002). The pooled odds ratios (ORs) showed that PD-L1 expression was associated with poor differentiation (OR = 3.47, 95% CI 1.37–8.77, *P* = 0.008) and right colon cancer (OR = 2.38, 95% CI 1.57–3.60, *P* < 0.0001). However, the expression of PD-L1 was independent of gender, age, tumor size, tumor stage, lymph node metastasis, and tumor-node metastasis stage.

**Conclusion:** This meta-analysis indicated that a high level of PD-L1 expression might be a biomarker for a poor prognosis in CRC patients. This information may be helpful for clinicians to stratify CRC patients for anti-PD-1/PD-L1 therapy, particularly patients with microsatellite instability high (MSI-H).

## Introduction

Globally, colorectal cancer (CRC) is the third leading cause of cancer (Siegel et al., 2017). Although cancer screening programs and the standardization of preoperative and postoperative care have reduced mortality associated with a CRC diagnosis (Welch and Robertson, 2016), CRC is still a leading cause of cancer-related deaths worldwide, for it has a poor prognosis in its malignant stages and recurrence is common. Therefore, it is essential to identify new biomarkers to improve clinical decision-making and patient outcomes.

As one of the most possible newly biomarkers to evaluate cancer patients' outcomes, programmed death 1 (PD-1) is an immune-inhibitory receptor that is expressed on the surface of activated T cells as a result of persistent inflammatory stimuli (Inaguma et al., 2016; Zou et al., 2016). PD-L1 is expressed by T and B cells, macrophages and dendritic cells and its expression implies a weakened host immune response and consequent a poor prognosis (Hansen et al., 2009). The binding of PD-L1 to PD-1 can attenuate the cellular immune response by reducing T cells apoptosis or exhaustion. Blockade of the PD-1/PD-L1 pathway with monoclonal antibodies is a highly promising therapy and prominent clinical benefits of this checkpoint-blockade were observed in recent clinical trials (Zheng and Zhou, 2015; Wang et al., 2018).

Positive PD-L1 expression has been associated with significantly poor prognoses; however, studies evaluating the prognostic significance of PD-L1 expression in CRC are limited and remain controversial. Therefore, we conducted a comprehensive meta-analysis to evaluate the clinicopathological and prognostic significance of PD-L1 expression in CRC patients.

## Materials and Methods

### Literature Search

Two authors (M. Z. He and Y. Y. Zhou) independently conducted comprehensive literature searches of published articles using the Medline/PubMed, Embase, Cochrane Library, WoS and Scopus databases. The endpoint for search items was July 21, 2018. The following keywords were used: (“colorectal” OR “colorectum” OR “colon” OR “Rectum” OR “Rectal” OR “large intestine”) AND (“adenocarcinoma?” OR “tumor?” OR “neoplasm?” OR “carcinoma?” OR “cancer?” OR “malignant”) AND (“Programmed Cell Death 1 Receptor” OR “CD279 Antigen” OR “PD-1” OR “B7-H1 Antigen” OR “Programmed Cell Death 1 Ligand 1” OR “PD-L1 “OR “CD 274”). Titles and abstracts were screened through NoteExpress and any discrepancies were resolved by mutual discussion.

### Eligibility Criteria

The criteria for inclusion were: (1) All patients were histologically confirmed as having CRC and had not received adjuvant chemotherapy before surgery; (2) PD-L1 expression was detected by immunohistochemistry (IHC); (3) Studies showed a correlation between PD-L1 expression with clinicopathological features and/ or prognoses; (4) Articles were published as a full paper in English. The criteria for exclusion were: (1) Case reports, reviews and letters; (2) The main content did not evaluate the relationship of PD-L1 expression with clinicopathological features and/ or prognoses; (3) duplications and studies without eligible data. When duplicate publications were identified, only the article with the newest and most comprehensive information was included.

### Data Extraction and Quality Assessment

The following information from the included articles was extracted by two reviewers (M. Z. He and Y. Y. Zhou): name of the first author, year of publication, country of origin, tumor type, number of cases, staining method, cut-off values, PD-L1 positive expression, clinicopathological parameters, outcome, and quality assessment score. Any disagreements between the two reviewers were resolved by consensus involving a third reviewer (Y. Li). Outcome parameters comprised OS, DFS and recurrence-free survival (RFS). The HRs and 95% confidence intervals (CIs) were evaluated for outcome parameters. If the HRs were not available, we extracted data from survival curves or contacted the corresponding authors.

According to the Newcastle-Ottawa Quality Assessment (NOS), a quality assessment was independently carried out for the included articles by two authors (M.Z. He and Y. Y. Zhou). Discrepancies in scoring were resolved by discussion and consensus. The NOS consists of the following three parameters of quality: selection, comparability and outcome. The maximum NOS score is nine points, with studies scoring greater than six considered to be of high quality (Stang, 2010).

### Statistical Methods

Pooled HRs and 95% CIs were calculated to evaluate the association between PD-L1 positive expression with OS, DFS, RFS and clinicopathological parameters. Heterogeneity among studies was evaluated using the Chi-squared test and *I*^2^. A random-effects model was used when there was evidence of significant heterogeneity (*I*^2^ > 50% or *P*-value < 0.1). In all other cases, a fixed-effects model was used. Potential publication bias was assessed through Egger's and Begg's tests. The statistical analysis was conducted using Review Manager Version 5.3 (Revman the Cochrane Collaboration; Oxford, England) and STATA version 14 (Stata Corporation; College Station, TX, USA). All *P-*values and 95% CIs were two-sided, and *P*-values < 0.05 were considered to be statistically significant.

## Results

### Search Results and Study Characteristics

**Figure 1:**
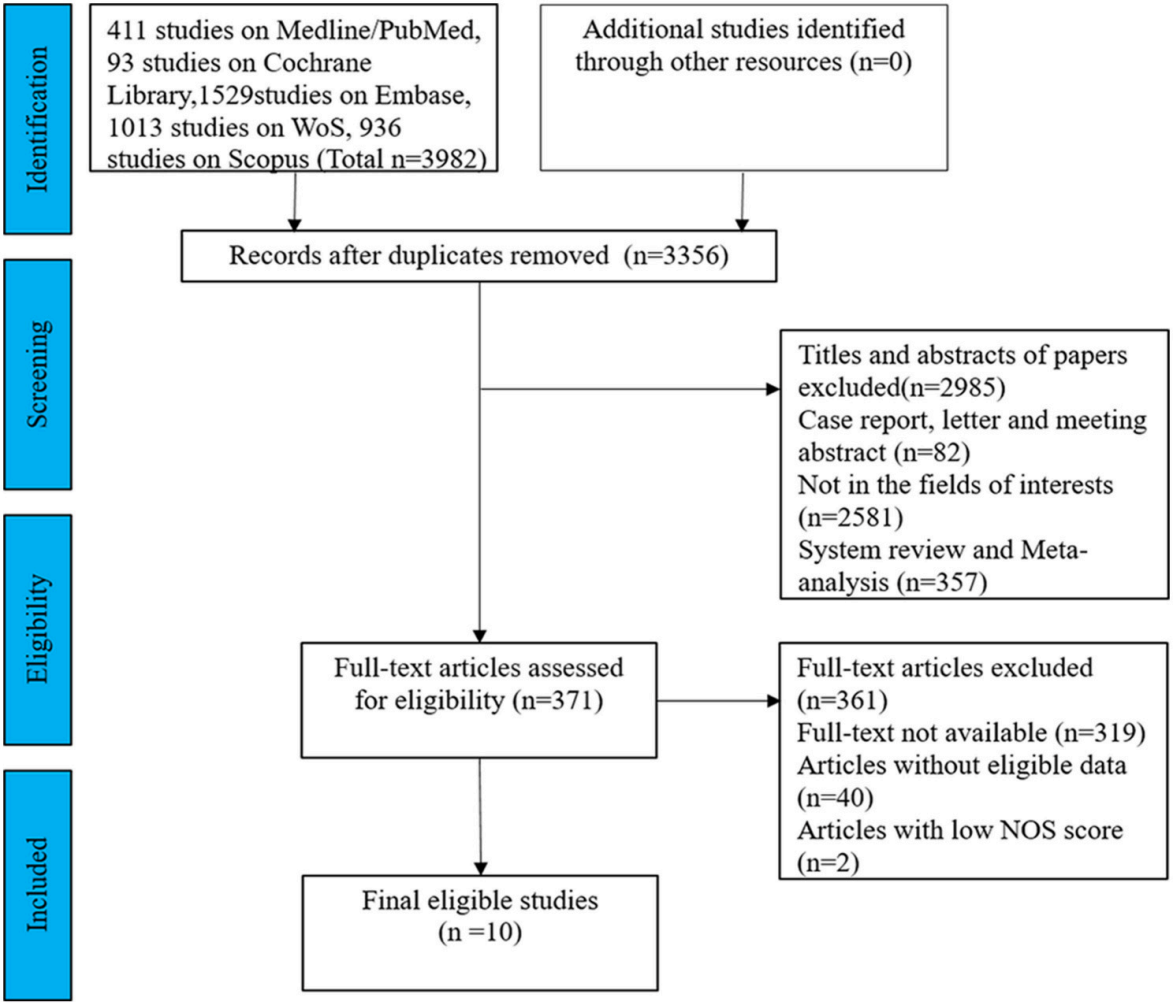
Flow diagram of the study selection process in this meta-analysis.

After exclusion of 626 duplicates, 3,356 articles about PD-1/PD-L1 in colorectal cancer were identified from a primary system literature search in the Medline/PubMed, Embase, Cochrane Library, WoS, and Scopus databases. The titles and abstracts of the remaining articles were screened, and 2,985 records were rejected because they were case reports, letters, meeting, reviews or not in the fields of interests. We read 371 records for further assessment. Among them, 319 full-text articles were not available, another 40 lacked eligible data, and two scored lower than 6 on the NOS. Finally, 10 articles were included in this meta-analysis. A flowchart of the literature selection is shown in Figure 1.

The characteristics of the 10 included studies are listed in Table 1. These included studies were generally of high quality, with NOS scores ranging from six to eight. All 10 studies were retrospective and published between 2013 and 2018. In total, 10 studies comprising 2,131 patients were included in the pooled analysis and all selected studies used IHC assays to evaluate PD-L1 expression in tumor cells and /or TILs. Each article had an independent cut-off value used to define the criterion for PD-L1 positive. Six studies provided OS data (Shi et al., 2013; Zhu et al., 2015; Li et al., 2016; Enkhbat et al., 2018; Lee S. J. et al., 2018; Liu et al., 2018), three studies included DFS data (Enkhbat et al., 2018; Lee K. S. et al., 2018; Lee S. J. et al., 2018) and three studies included RFS data (Lee et al., 2016; Wang et al., 2016, 2017). In addition, HRs and 95% CIs were abstracted directly from the 10 included studies.

**Table 1:** Main characteristics of the studies included for meta-analysis.

**References**	**Country**	**No**.	**Tumor Histology**	**Stage**	**Technique**	**Cut-off**	**PD-L1(+) (%)**	**Outcome**	**HR estimation (95% CI)**	**Quality assessment (score)**
Shi et al., 2013	China	143	CRC	I-IV	IHC	Moderate or intense staining	TC: 64/143(44.8)	OS	TC: 2.77(1.05–2.99)	6
Zhu et al., 2015	China	120	SAC	NA	IHC	IRS≥ 4	TC: 28/120(23.3)	OS	TC: 2.30(1.13–4.68)	7
Lee et al., 2016	USA	395	CRC	I-IV	IHC+TMA	≥10% and intense staining	TC: 19/394(4.8)	RFS	TC: 22.86(1.99–263.21)	6
Wang et al., 2016	Switzerland	262	CRC	II-III	IHC+TMA	≥5%	TILs: 54/262(20.6)	RFS	TC: 1.90(0.88–4.14) TILs: 1.83(1.09–3.05)	7
Wang et al., 2017	China	254	CRC	II-III	IHC+TMA	NA	TILs: 89/254(35.0)	RFS	TILs: 1.74(1.02–2.98)	6
Li et al., 2016	China	356 (TCGA)	CRC	NA	IHC+TMA	IRS> 4	TC: 301/356(84.6)	OS	TC: 0.63 (0.33–1.18)	8
Enkhbat et al., 2018	Japan	116	CRC	II-III	IHC	IRS≥ 4	TC: 52/116(44.8)	OS DFS	OS: TC:3.87(1.19–12.57) DFS: TC:1.91(0.81–4.52)	6
Liu et al., 2018	China	60	mCRC	NA	IHC	IS≥ 3	TC: 26/60(43.3)	OS	TC:0.28(0.08–0.99)	6
Lee K. S. et al., 2018	South Korea	89	CC(MSI)	I-III	IHC	≥5%	TILs: 56/89(62.9)	DFS	TILs:0.33(0.11–0.80)	6
Lee S. J. et al., 2018	South Korea	336	CRC	0-IV	IHC+TMA	≥1%	TC: 15/336(9.4)	OS DFS	OS: TC:3.78(1.45–9.90) DFS: TC:3.50(1.46–8.41)	7

*CRC, colorectal cancer; SAC, serrated adenocarcinoma; mCRC, metastatic colorectal cancer; CC, colon cancer; MSI, microsatellite instability; IHC, immunohistochemistry; NA, not available; TMA, tissue microarray; OS, overall survival; HR, hazard ratio; TC, tumor cell; TILs, tumor-infiltrating lymphocytes; IRS, Immunoreactivity score; IS, Immunoscore; DFS, disease-free survival; RFS, recurrence-free survival*.

### Association Between PD-L1 Expression and Prognostic Parameters

**Figure 2:**
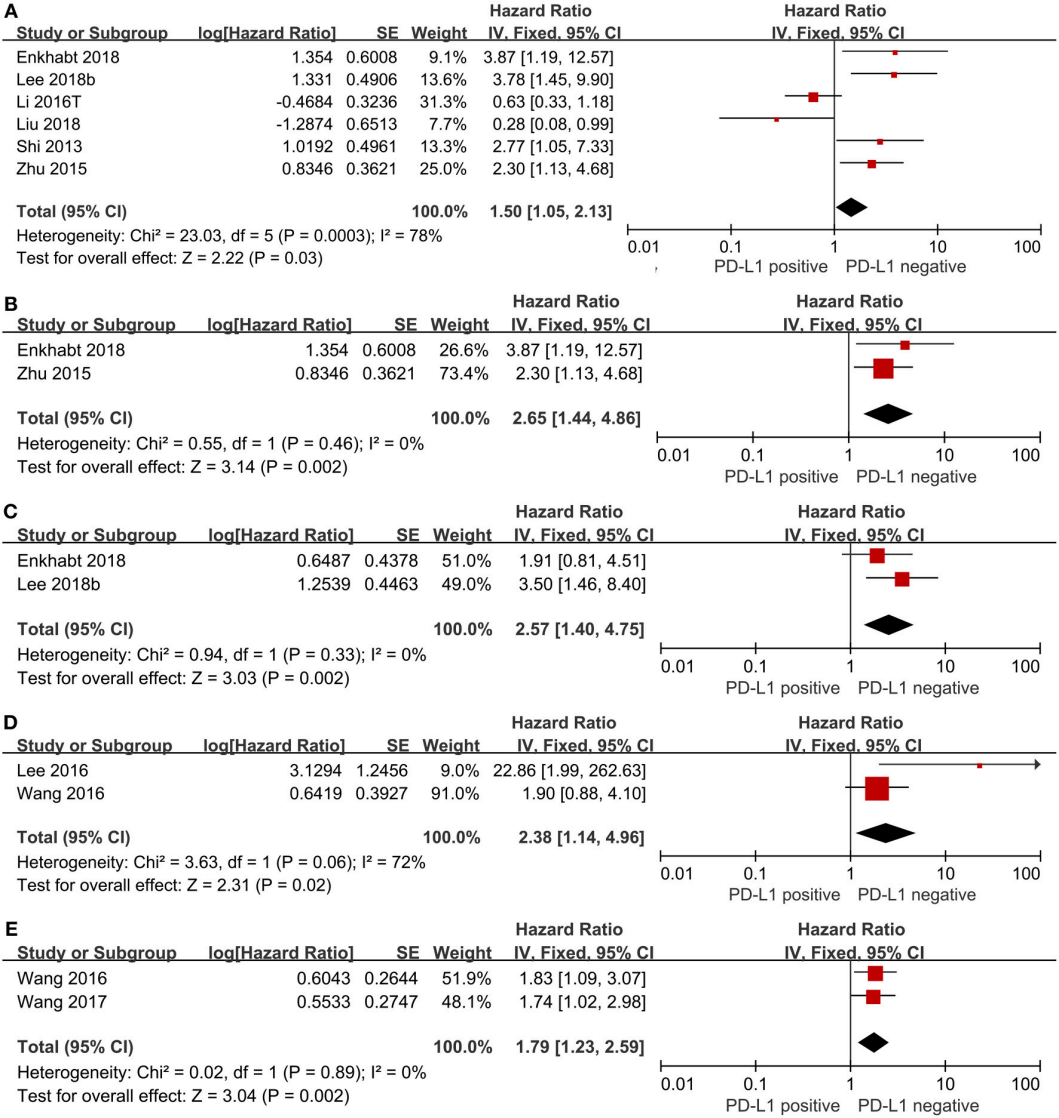
Forest plot of 10 studies evaluating the association between PD-L1 expression and prognostic parameters in CRC patients (**A**: OS in TC; **B**: IRS≥4 as cut-off value; **C**: DFS in TC; **D**: RFS in TC; **E**: RFS in TILs).

We evaluated the association between PD-L1 expression and prognostic parameters (OS, DFS and RFS). The pooled HR for OS in TC from six studies, involving 1,131 patients, showed that PD-L1 expression was significantly associated with poor OS in CRC (HR = 1.50, 95%CI 1.05–2.13, *P* = 0.03; see Figure 2A). When we took Immunoreactivity score (IRS) ≥ 4 as the cut-off value, we found shorter survival in the PD-L1 positive group (HR = 2.65, 95%CI 1.44–4.86, *P* = 0.002; see Figure 2B). The pooled HR for DFS in TC with 452 patients indicated that PD-L1 expression was significantly associated with shorter DFS (HR = 2.57, 95%CI 1.40–4.75, *P* = 0.002; see Figure 2C). The pooled HR for RFS in TC with 657 patients (HR = 2.38, 95%CI 1.14–4.96, *P* = 0.02; see Figure 2D) as well as the pooled HR for RFS in tumor-infiltrating lymphocytes (TILs) with 516 CRC patients (HR = 1.79, 95%CI 1.23–2.95, *P* = 0.002; see Figure 2E) showed that PD-L1 expression was significantly associated with poor RFS both in TC and TILs.

### Association Between PD-L1 Expression and Clinicopathological Characteristics

#### Gender

**Figure 3:**
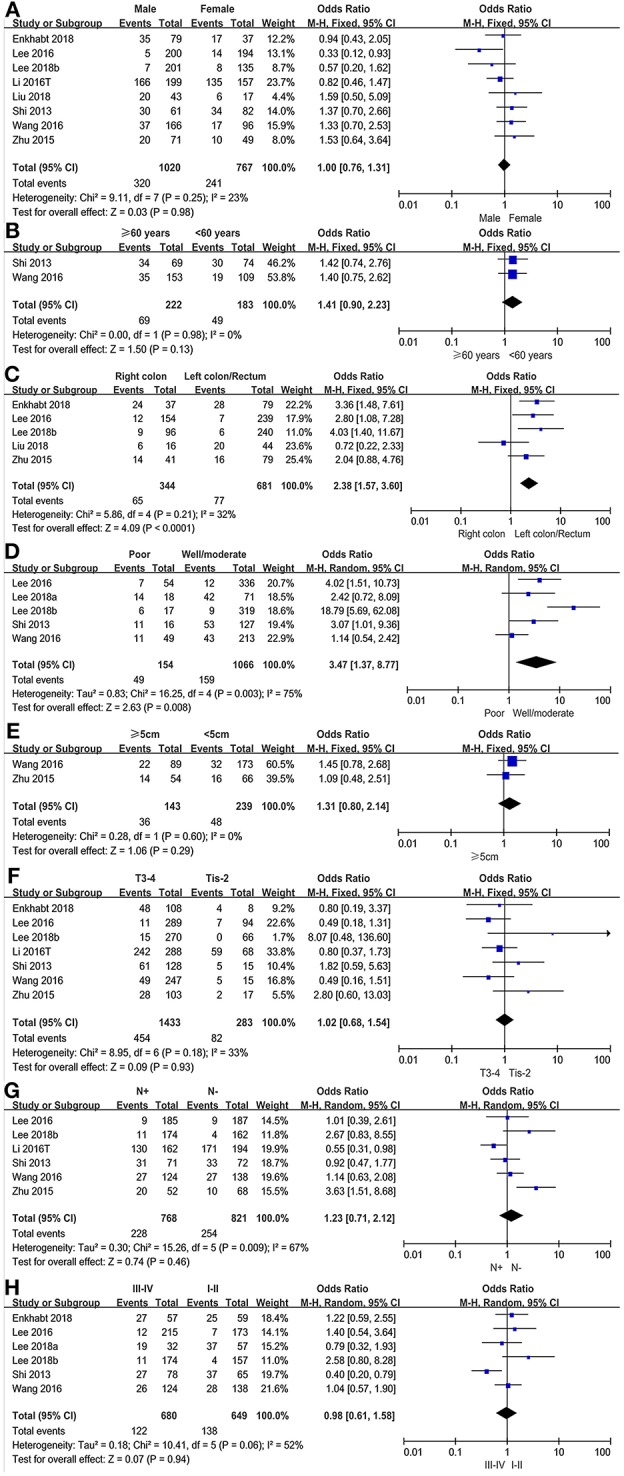
Forest plots for the association of PD-L1 expression with clinicopathological features in CRC patients (**A**: gender; **B**: age; **C**: cancer location; **D**: differentiation; **E**: tumor size; **F**: T stage; **G**: lymph node metastasis; **H**: TNM stage).

The association between PD-L1 expression and gender was evaluated in eight studies, comprising 3,477 patients. 320(31.37%) of 1,020 male patients and 241(31.42%%) of 767 female patients were PD-L1 expression positive. The pooled OR showed that there was no significant association found between PD-L1 expression and gender (OR = 1.00, 95%CI 0.76–1.31, *P* = 0.98; see Figure 3A).

#### Age

We evaluated the association between PD-L1 expression and age in a total of 405 patients from two studies. 49 (26.78%) of 183 younger patients (<60 years of age) were PD-L1 expression positive and 69 (31.08%) of 222 older patients (≥60 years of age) were PD-L1 expression positive. There was no significant association found between PD-L1 expression and age (OR = 1.41, 95% CI 0.90–2.23, *P* = 0.13; see Figure 3B).

#### Cancer Location

The association between PD-L1 expression and cancer location was analyzed in six studies with a population of 1,025 patients. Of 344 right colon cancer patients, 65 (18.90%) were PD-L1 expression positive, while 77(11.31%) in 681 left colon/rectum cancer patients. The pooled OR showed a significant association between PD-L1 expression and cancer location (OR = 2.38, 95% CI 1.57–3.60, *P* < 0.0001; see Figure 3C).

#### Differentiation

Of 1,066 well/moderately differentiated tumors, 159 (14.92%) were PD-L1 expression positive. Of 154 poorly differentiated tumors, 49 (34.82%) were PD-L1 expression positive. The pooled OR showed that PD-L1 expression was significantly associated with differentiation based on pooled data from five studies (OR = 3.47, 95%CI 1.37–8.77, *P* = 0.008; see Figure 3D).

#### Tumor Size

Only two studies, including 382 colorectal cancer patients, analyzed the subgroup of tumor size based on the cut-off value of 5 cm. 36 (25.17%) of 143 patients with large tumors (≥5 cm) and 48 (20.01%) of 239 patients with small tumors (<5 cm) were PD-L1 expression positive. The pooled results carried out in a fixed effect model, showed that there was no significant association between PD-L1 expression and tumor size (OR = 1.31, 95%CI 0.80–2.14, *P* = 0.29; see Figure 3E).

#### T Stage

We evaluated the association between PD-L1 expression and T stage in 1,716 patients. Of 283 Tis-T2 stage patients, 82 (28.98%) were PD-L1 expression positive and 454 (31.68%) of 1,433 T3-T4 stage patients were PD-L1 expression positive. The pooled HR showed that there was no significant association between PD-L1 expression and T stage (OR = 1.02, 95%CI 0.68–1.54, *P* = 0.93; see Figure 3F).

#### Lymph Node Metastasis

The association between PD-L1 expression and lymph node metastasis was evaluated in six studies (1,589 patients). The pooled OR indicated that there was no significant association found between PD-L1 expression and lymph node metastasis (OR = 1.23, 95%CI 0.71–2.12, *P* = 0.46; see Figure 3G).

#### TNM Stage

Six studies, involving 1,329 patients, evaluated the association between PD-L1 expression and TNM stage in a fixed effects model. 138 (21.26%) of 649 stage I-II patients and 122 (17.94%) of 680 stage III-IV patients were PD-L1 expression positive. The pooled result showed no significant association found between PD-L1 expression and TNM stage (OR = 0.98, 95%CI 0.61–1.58, *P* = 0.94; see Figure 3H).

Heterogeneity was identified in the analysis of PD-L1 expression with cancer location (*P* = 0.73, *I*^2^ = 82%) and lymph node metastasis (*P* = 0.46, *I*^2^ = 67%). Therefore, a random effects model was used in the above analyses and other subgroup analyses were performed in a fixed effects model.

### Publication Bias

**Figure 4:**
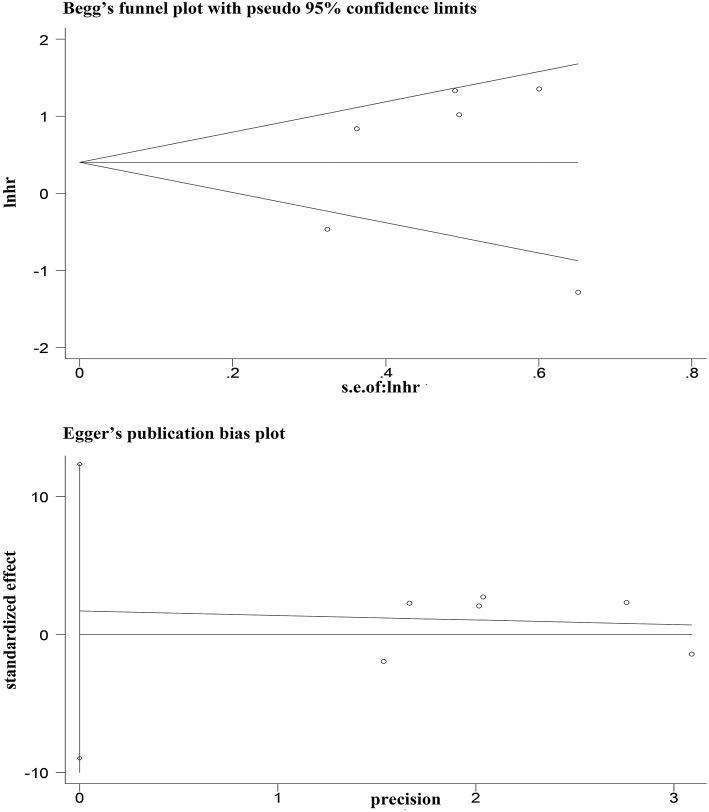
Egger's and Begg's funnel plot with 95% CI for OS publication bias in the included six studies.

**Figure 5:**
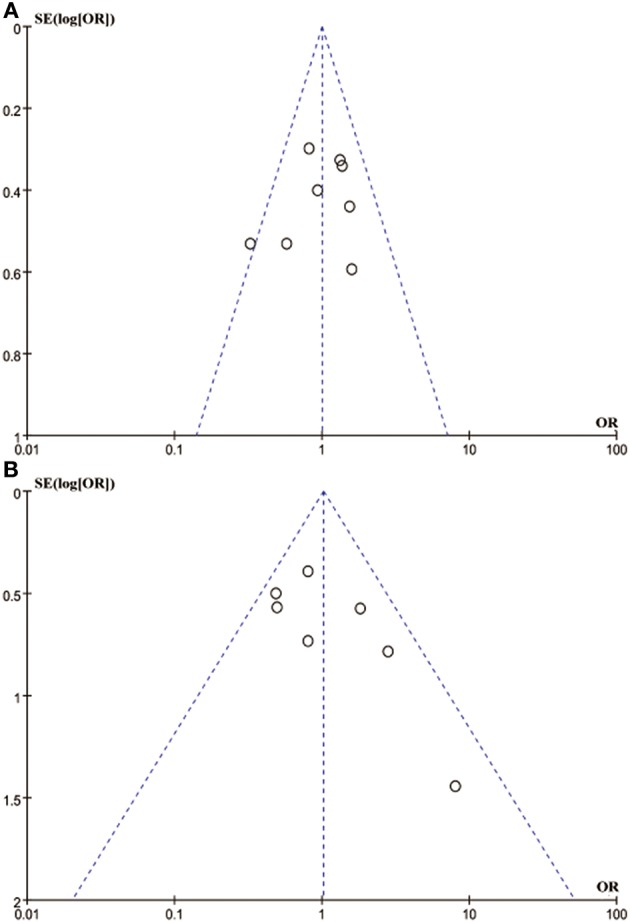
Funnel blot visualizing a potential publication bias of PD-L1 expression with gender and T stage (**A**: gender; **B**: T stage).

Egger's and Begg's tests showed that no publication bias influencing the HRs for OS was observed in the six studies (Figure 4). The *P*-values for these tests were 0.683 and 1.000, respectively. In addition, the funnel plots showed no publication bias for gender or T stage (Figure 5).

## Discussion

In the present meta-analysis of the clinicopathological and prognostic significance PD-L1 expression in CRC, we found that PD-L1 expression was significantly associated with poor OS in TC. In addition, the pooled results of RFS and DFS showed that PD-L1 expression was significantly correlated with unfavorable clinical outcomes. Poor differentiation and right colon CRC tumors suggested a poor prognosis. The expression of PD-L1 was independent of gender, age, tumor size, T stage, lymph node metastasis, and TNM stage. To our knowledge, this comprehensive meta-analysis is the first to evaluate the association of PD-L1 expression with clinicopathological characteristics and prognostic parameters in colorectal cancer.

During the process of study of selection, the study of Droeser et al. (2013) was excluded for it included unselected, non-consecutive, primary, sporadic colorectal cancers, and the data of the included articles in this meta-analysis were satisfied with a more rigorous standards, which excluded the patients receiving adjuvant chemotherapy before surgery, diagnosis of gastrointestinal stromal tumor or lymphoma, diagnosis with additional cancers. It is well-known that accurate results were based on the rigorous exclusion criteria in retrospective study. Among the OS data in six included studies, one study showed contradictory results showing that PD-L1 positive expression was significantly associated with better OS. This study was not the only one to report a positive prognostic impact of PD-L1 expression. Sabatier et al. (Schalper et al., 2014) evaluated PD-L1 expression in 5,454 breast cancer cases and found that positive PD-L1 expression was associated with better metastasis-free survival and improved response to chemotherapy. However, the pooled result showed a significant correlation of PD-L1 expression and poor prognostic outcomes was supported by other articles reporting poorer outcomes in renal cell carcinoma, non-small cell lung cancer (Wang et al., 2015) and osteosarcoma (Lussier et al., 2015). This was because of the complex function of PD-L1 in the initiation and growth of CRC.

PD-L1 is upregulated by many inflammatory mediators and cytokines (Keir et al., 2006, 2008; Okazaki and Honjo, 2006) and PD1/PD-L1 binding can induce activated T cell apoptosis, exhaustion, and interleukin-10(IL-10) expression as a negative feedback system (Zou et al., 2016). Moreover, PD-L1 expression can help tumor cells to evade immunosurveillance and enhance the function of Tregs in CRC (Lu et al., 2011; Toh et al., 2016). However, MSI tumors in CRC display high infiltration with CD8+ cytotoxic T lymphocytes (CTLs) and activated Th1 cells, which may contribute to better survival (Gubin et al., 2014). MSI tumors are also counterbalanced by upregulating expression of multiple immune checkpoints (Angelova et al., 2015; Becht et al., 2016), such as CTL-associated antigen 4 (CTLA4), PD-1, PD-L1 and indoleamine 2,3-dioxygenase 1 (IDO1). Upregulated after T cell activation, PD-1 declines when an antigen is cleared. While PD-1 expression remains elevated, as in CRC cancer, T cells enter a state of exhaustion or anergy (Xiao and Freeman, 2015). A study found that *Fusobacterium* species could evade the high load of neoantigens in MSI colorectal cancer (Tahara et al., 2014). And these species may facilitate upregulation of PD-L1 and lead to poor survival (Kostic et al., 2013). Considering the dynamic changes of PD-L1 expression, our results showing that PD-L1 expression was significantly associated with poor prognoses appear more credible.

We also noticed a recently literature make a contradictory conclusion with our study. This study considered that no significant differences founded in colorectal cancer-specific or overall survival by Tumor Immunity in the MicroEnvironment (TIME) subtypes (Hamada et al., 2018). We found that the primary data of their study were too old, as one cohort was from 1986 to 1992 and the other was from 1986 to 2004 (Giovannucci et al., 1995; Wark et al., 2009). While, our primary data were carried out from 2006 to 2016. The discrepancies between Hamada et al. (2018) and our study might reflect the different storage time of tissue sections. Reports by Bertheau et al. (1998) and Jacobs et al. (1996), who investigated the loss of immunoreactivity for a panel of antibodies in breast carcinomas, neuroendocrine tumors and lymphomas, indicated that for the majority of epitopes tested there is a time-dependent substantial loss in stored tissue slides. CRC develops via sequential genetic and epigenetic alterations of TCs, and is influenced by tumor-host interactions. Because CRC patients easily developed local recurrences and distant metastases within 5 years after surgical treatment and CRC has typical immune subgroups (Dienstmann et al., 2017), researchers found that immunotherapy is able to reach center stage in the field of second-line therapy in oncology treatment (Topalian et al., 2012; Hon et al., 2018). As one of the types of CRC, high microsatellite instability (MSI-H) can gather TILs and upregulate PD-L1 expression in tumor cells (Herbst et al., 2014). Currently, PD-L1 expression on TCs is considered as an immune-tolerance mechanism of carcinoma, because it can attract PD-1 expressing immune-inhibitory TILs. However, little is known about the complex interrelationship among PD-L1 expression, TILs, and major tumor molecular features. PD-L1 promoter methylation (mPD-L1) was significantly correlated with poor PD-L1 mRNA expression, indicating that PD-L1 expression might be regulated by mPD-L1 on a cellular level in CRC (Goltz et al., 2016). However, this study was not available to provide data on PD-L1 protein expression and there was a study had published a proteomic characterization of the cohort, showing that protein abundance could not be reliably predicted from DNA- or RNA-level measurements (Zhang et al., 2014). Previous studies have shown a significant correlation of PD-L1 expression with OS in melanoma (Robert et al., 2015), breast cancer (Zhang et al., 2017), renal cell carcinoma (Motzer et al., 2014), and non-small cell lung cancer (Zhang et al., 2015), and observed prominent clinical benefits of PD-1/PD-L1 checkpoint blockades in these carcinoma patients. Although previous trials have suggested no role for immunotherapy in patients with CRC, recent studies have demonstrated that MSI-H in CRC did benefit (Kwak et al., 2016; Overman et al., 2017). Therefore, we investigated the relationship between the expression of PD-L1 and clinicopathological factors, and the results showed that poor differentiation and right colon location in CRC were PD-L1 expression positive. In addition, poor differentiation and right colon location in CRC were also significantly correlated with poor prognoses, which were more likely to be MSI-H. Thus, our study provided a scientific rationale and direct support for clinicians to select MSI-H CRC patients for anti-PD-1/PD-L1 immunotherapy.

This study provided moderate evidence to evaluate the association of PD-L1 expression with prognostic outcomes and clinicopathological factors. However, there were some limitations. Firstly, only six included studies evaluated the association of PD-L1 expression with OS. Although the sample sizes of RFS and DFS were relatively small, their results should have alleviated some of these concerns. Secondly, the cut-off values determining positive and negative PD-L1 expression and antibodies for PD-L1 varied among the included studies. Thus, the subgroup of IRS ≥ 4 had reduced heterogeneity and addressed some of these concerns. Thirdly, only articles published in English were included. Accordingly, to address these limitations, a large multicenter study with uniform evaluation methods (the same antibody and cut-off for positive PD-L1 expression) may be helpful to attain results that are more accurate. Despite the above limitations, the present meta-analysis demonstrated the association of PD-L1 expression with prognostic outcomes and clinicopathological factors. The findings of this study may lead to improvements in the outcomes of anti-PD-1/PD-L1 therapy through stratifying patients in a more appropriate manner.

## Conclusion

In conclusion, our results showed that PD-L1 positive expression might be a new biomarker for poor prognosis in CRC. This information may be helpful for clinicians to stratify CRC patients for anti-PD-1/PD-L1 therapy, especially patients with MSI-H. Well-designed and high-quality studies with uniform evaluation methods are needed to confirm the association of PD-L1 expression in CRC.

## Author Contributions

YL, MH, and LX designed this study. YZ and MH screened identified studies and extracted data. Disagreements were resolved by discussion with YL and YZ performed the statistical analyses. MH and YZ prepared the figures and tables. YL, MH, and LX reviewed the results, interpreted the data, and wrote the manuscript. All authors have read and approved the final version of this manuscript.

### Conflict of Interest Statement

The authors declare that the research was conducted in the absence of any commercial or financial relationships that could be construed as a potential conflict of interest.

## Abbreviations

PD-L1: programmed death-ligand 1
CRC: colorectal cancer
WoS: wet of science
HR: hazard ratio
TCs: tumor cells
OS: overall survival
DFS: disease-free survival
ORs: odds ratios
T stage: tumor stage
TNM: tumor-node-metastasis
MSI-H: microsatellite instability high
PD-1: programmed death 1
IHC: immunohistochemistry
CIs: confidence intervals
NOS: newcastle-ottawa quality assessment
IRS: immunoreactivity score
TILs: tumor-infiltrating lymphocytes
CTLs: CD8+ cytotoxic T lymphocytes
CTLA4: CTL-associated antigen 4
IDO1: indoleamine 2,3-dioxygenase 1
TIME: tumor immunity in the microEnvironment
mPD-L1: PD-L1 promoter methylation.

## References

[B1] Angelova M. Charoentong P. Hackl H. Fischer M. L. Snajder R. Krogsdam A. M.. (2015). Characterization of the immunophenotypes and antigenomes of colorectal cancers reveals distinct tumor escape mechanisms and novel targets for immunotherapy. Genome Biol. 16:64. 10.1186/s13059-015-0620-6 25853550 PMC4377852

[B2] Becht E. de Reyniès A. Giraldo N. A. Pilati C. Buttard B. Lacroix L.. (2016). Immune and stromal classification of colorectal cancer is associated with molecular subtypes and relevant for precision immunotherapy. Clin. Cancer Res. 22, 4057–4066. 10.1158/1078-0432.CCR-15-2879 26994146

[B3] Bertheau P. Cazalshatem D. Meignin V. de Roquancourt A. Vérola O. Lesourd A.. (1998). Variability of immunohistochemical reactivity on stored paraffin slides. J. Clin. Pathol. 51, 370–374. 10.1136/jcp.51.5.370 9708203 PMC500697

[B4] Dienstmann R. Vermeulen L. Guinney J. Kopetz S. Tejpar S. Tabernero J. (2017). Consensus molecular subtypes and the evolution of precision medicine in colorectal cancer. Nat. Rev. Cancer 17:79 10.1038/nrc.2016.126 28050011

[B5] Droeser R. A. Hirt C. Viehl C. T. Frey D. M. Nebiker C. Huber X.. (2013). Clinical impact of programmed cell death ligand 1 expression in colorectal cancer. Eur. J. Cancer 49:233. 10.1016/j.ejca.2013.02.015 23478000

[B6] Enkhbat T. Nishi M. Takasu C. Yoshikawa K. Jun H. Tokunaga T.. (2018). Programmed Cell death ligand 1 expression is an independent prognostic factor in colorectal cancer. Anticancer Res. 38, 3367–3373. 10.21873/anticanres.12603 29848685

[B7] Giovannucci E. Ascherio A. Rimm E. B. Colditz G. A. Stampfer M. J. Willett W. C. (1995). Physical activity, obesity, and risk for colon cancer and adenoma in men. Ann. Intern. Med. 122:327. 10.7326/0003-4819-122-5-199503010-00002 7847643

[B8] Goltz D. Gevensleben H. Dietrich J. Dietrich D. (2016). PD-L1 (CD274) promoter methylation predicts survival in colorectal cancer patients. Onco Immunol. 6:e1257454. 10.1080/2162402X.2016.1257454 28197377 PMC5283627

[B9] Gubin M. M. Zhang X. Schuster H. Caron E. Ward J. P. Noguchi T. (2014). Checkpoint blockade cancer immunotherapy targets tumor-specific mutant antigens. Nature 515, 577–581. 10.1038/nature13988 25428507 PMC4279952

[B10] Hamada T. Soong T. R. Masugi Y. Kosumi K. Nowak J. A. da Silva A.. (2018). TIME (Tumor Immunity in the MicroEnvironment) classification based on tumor CD274 (PD-L1) expression status and tumor-infiltrating lymphocytes in colorectal carcinomas. Onco Immunol.7:e1442999. 10.1080/2162402X.2018.1442999 29900052 PMC5993482

[B11] Hansen J. D. Du Pasquier L. Lefranc M. P. Lopez V. Benmansour A. Boudinot P. (2009). The B7 family of immunoregulatory receptors: a comparative and evolutionary perspective. Mol. Immunol. 46, 457–472. 10.1016/j.molimm.2008.10.007 19081138

[B12] Herbst R. S. Soria J. C. Kowanetz M. Fine G. D. Hamid O. Gordon M. S.. (2014). Predictive correlates of response to the anti-PD-L1 antibody MPDL3280A in cancer patients. Nature 515:563. 10.1038/nature14011 25428504 PMC4836193

[B13] Hon K. W. Abu N. Ab Mutalib N. S. Jamal R. (2018). miRNAs and lncRNAs as predictive biomarkers of response to FOLFOX therapy in colorectal cancer. Front. Pharmacol. 9:846. 10.3389/fphar.2018.00846 30127741 PMC6088237

[B14] Inaguma S. Lasota J. Wang Z. Felisiak-Golabek A. Ikeda H. Miettinen M. (2016). Clinicopathologic profile, immunophenotype, and genotype of CD274 (PD-L1)-positive colorectal carcinomas. Mod. Pathol. 30:278. 10.1038/modpathol.2016.185 27813511 PMC7900912

[B15] Jacobs T. W. Prioleau J. E. Stillman I. E. Schnitt S. J. (1996). Loss of tumor marker-immunostaining intensity on stored paraffin slides of breast cancer. J. Natl. Cancer Inst. 88, 1054–1059. 10.1093/jnci/88.15.1054 8683636

[B16] Keir M. E. Butte M. J. Freeman G. J. Sharpe A. H. (2008). PD-1 and its ligands in tolerance and immunity. Ann. Rev. Immunol. 26, 677–704. 10.1146/annurev.immunol.26.021607.090331 18173375 PMC10637733

[B17] Keir M. E. Liang S. C. Guleria I. Latchman Y. E. Qipo A. Albacker L. A. (2006). Tissue expression of PD-L1 mediates peripheral T cell tolerance. Exp. Med. 2203, 883–895. 10.1084/jem.20051776 PMC2118286 16606670

[B18] Kostic A. D. Chun E. Robertson L. Glickman J. N. Gallini C. A. Michaud M.. (2013). Fusobacterium nucleatum potentiates intestinal tumorigenesis and modulates the tumor-immune microenvironment. Cell Host Microbe 14, 207–215. 10.1016/j.chom.2013.07.007 23954159 PMC3772512

[B19] Kwak Y. Koh J. Kim D. W. Kang S. B. Kim W. H. Lee H. S. (2016). Immunoscore encompassing CD3+ and CD8+ T cell densities in distant metastasis is a robust prognostic marker for advanced colorectal cancer. Oncotarget 7, 81778–81790. 10.18632/oncotarget.13207 27835889 PMC5348429

[B20] Lee K. S. Kim B. H. Oh H. K. Kim D. W. Kang S. B. Kim H.. (2018). Programmed cell death ligand-1 protein expression and CD274/PD-L1 gene amplification in colorectal cancer: implications for prognosis. Cancer Sci. 109, 2957–2969. 10.1111/cas.13716 29949671 PMC6125474

[B21] Lee L. H. Cavalcanti M. S. Segal N. H. Hechtman J. F. Weiser M. R. Smith J. J.. (2016). Patterns and prognostic relevance of PD-1 and PD-L1 expression in colorectal carcinoma. Modern Pathol. 29:1433. 10.1038/modpathol.2016.139 27443512 PMC5083129

[B22] Lee S. J. Jun S. Y. Lee I. H. Kang B. W. Park S. Y. Kim H. J.. (2018). CD274, LAG3, and IDO1 expressions in tumor-infiltrating immune cells as prognostic biomarker for patients with MSI-high colon cancer. J. Cancer Res. Clin. Oncol. 144, 1005–1014. 10.1007/s00432-018-2620-x 29520442 PMC11813403

[B23] Li Y. Lei L. Dai W. Cai G. Xu Y. Li X.. (2016). Prognostic impact of programed cell death-1 (PD-1) and PD-ligand 1 (PD-L1) expression in cancer cells and tumor infiltrating lymphocytes in colorectal cancer. Mol. Cancer 15:55. 10.1186/s12943-016-0539-x 27552968 PMC4995750

[B24] Liu R. Peng K. Yu Y. Liang L. Xu X. Li W.. (2018). Prognostic value of immunoscore and PD-L1 expression in metastatic colorectal cancer patients with different RAS status after palliative operation. BioMed Res. Int. 2018, 1–8. 10.1155/2018/5920608 29662888 PMC5831827

[B25] Lu B. Chen L. Liu L. Zhu Y. Wu C. Jiang J.. (2011). T-cell-mediated tumor immune surveillance and expression of B7 co-inhibitory molecules in cancers of the upper gastrointestinal tract. Immunol. Res. 50, 269–275. 10.1007/s12026-011-8227-9 21717068

[B26] Lussier D. M. Johnson J. L. Hingorani P. Blattman J. N. (2015). Combination immunotherapy with α-CTLA-4 and α-PD-L1 antibody blockade prevents immune escape and leads to complete control of metastatic osteosarcoma. J. Immunother. Cancer 3:21. 10.1186/s40425-015-0067-z 25992292 PMC4437699

[B27] Motzer R. J. Rini B. I. McDermott D. F. Redman B. G. Kuzel T. M. Harrison M. R.. (2014). Nivolumab for metastatic renal cell carcinoma: results of a randomized phase II trial. J. Clin. Oncol. 33, 1430–1437. 10.1200/JCO.2014.59.0703 25452452 PMC4806782

[B28] Okazaki T. Honjo T. (2006). The PD-1-PD-L pathway in immunological tolerance. Trends Immunol. 27, 195–201. 10.1016/j.it.2006.02.001 16500147

[B29] Overman M. J. McDermott R. Leach J. L. Lonardi S. Lenz H. J. Morse M. A.. (2017). Nivolumab in patients with metastatic DNA mismatch repair-deficient or microsatellite instability-high colorectal cancer (CheckMate 142): an open-label, multicentre, phase 2 study. Lancet Oncol. 18:1182. 10.1016/S1470-2045(17)30422-9 28734759 PMC6207072

[B30] Robert C. Long G. V. Brady B. Dutriaux C. Maio M. Mortier L.. (2015). Nivolumab in previously untreated melanoma without BRAF mutation. N. Engl. J. Med. 372, 320–330. 10.1056/NEJMoa1412082 25399552

[B31] Schalper K. A. Velcheti V. Carvajal D. Wimberly H. Brown J. Pusztai L.. (2014). *In situ* tumor PD-L1 mRNA expression is associated with increased TILs and better outcome in breast carcinomas. Clin. Cancer Res. 20, 2773–2782. 10.1158/1078-0432.CCR-13-2702 24647569

[B32] Shi S. J. Wang L. J. Wang G. D. Guo Z. Y. Wei M. Meng Y. L.. (2013). B7-H1 Expression is associated with poor prognosis in colorectal carcinoma and regulates the proliferation and invasion of HCT116 colorectal cancer cells. PLoS ONE 8:e76012. 10.1371/journal.pone.0076012 24124529 PMC3790819

[B33] Siegel R. L. Miller K. D. Fedewa S. A. Ahnen D. J. Meester R. G. S. Barzi A.. (2017). Colorectal cancer statistics. CA Cancer J. Clin. 67, 104–117. 10.3322/caac.21395 28248415

[B34] Stang A. (2010). Critical evaluation of the Newcastle-Ottawa scale for the assessment of the quality of nonrandomized studies in meta analyses. Eur. J. Epidemiol. 25, 603–605. 10.1007/s10654-010-9491-z 20652370

[B35] Tahara T. Yamamoto E. Suzuki H. Maruyama R. Chung W. Garriga J.. (2014). Fusobacterium in colonic flora and molecular features of colorectal carcinoma. Cancer Res. 74, 1311–1318. 10.1158/0008-5472.CAN-13-1865 24385213 PMC4396185

[B36] Toh J. W. T. de Souza P. Lim S. H. Singh P. Chua W. Ng W.. (2016). The potential value of immunotherapy in colorectal cancers: review of the evidence for programmed death-1 inhibitor therapy. Clin. Colorec. Cancer 15:285. 10.1016/j.clcc.2016.07.007 27553906

[B37] Topalian S. L. Hodi F. S. Brahmer J. R. Gettinger S. N. Smith D. C. McDermott D. F.. (2012). Safety, activity, and immune correlates of anti–PD-1 antibody in cancer. N. Engl. J. Med. 366, 2443–2454. 10.1056/NEJMoa1200690 22658127 PMC3544539

[B38] Wang A. Wang H. Y. Liu Y. Zhao M. C. Zhang H. J. Lu Z. Y.. (2015). The prognostic value of PD-L1 expression for non-small cell lung cancer patients: a meta-analysis. Eur. J. Surg. Oncol. 41, 450–456. 10.1016/j.ejso.2015.01.020 25682184

[B39] Wang L. Liu Z. Fisher K. W. Ren F. Lv J. Davidson D. D.. (2017). Prognostic value of programmed death ligand 1, p53, and Ki-67 in patients with advanced stage colorectal cancer. Hum. Pathol.71, 20–29. 10.1016/j.humpath.2017.07.014 28782638

[B40] Wang L. Ren F. Wang Q. Baldridge L. A. Monn M. F. Fisher K. W.. (2016). Significance of programmed death ligand 1 (PD-L1) immunohistochemical expression in colorectal cancer. Mol. Diagn. Ther. 20, 175–181. 10.1007/s40291-016-0188-1 26891728

[B41] Wang Y. Wang H. Yao H. Li C. Fang J. Y. Xu J. (2018). Regulation of PD-L1: emerging routes for targeting tumor immune evasion. Front. Pharmacol. 19:536 10.3389/fphar.2018.00536 PMC5992436 29910728

[B42] Wark P. A. Wu K. van 't Veer P. Fuchs C. F. Giovannucci E. L. (2009). Family history of colorectal cancer: a determinant of advanced adenoma stage or adenoma multiplicity? Int. J. Cancer 125, 413–420. 10.1002/ijc.24288 19358277 PMC2914547

[B43] Welch H. G. Robertson D. J. (2016). Colorectal cancer on the decline - why screening can't explain it all. N. Engl. J. Med. 374:1605. 10.1056/NEJMp1600448 27119236

[B44] Xiao Y. Freeman G. J. (2015). The microsatellite instable subset of colorectal cancer is a particularly good candidate for checkpoint blockade immunotherapy. Cancer Discov. 5:16. 10.1158/2159-8290.CD-14-1397 25583798 PMC4295637

[B45] Zhang B. Wang J. Wang X. Zhu J. Liu Q. Shi Z.. (2014). Proteogenomic characterization of human colon and rectal cancer. Nature 513, 382–387. 10.1038/nature13438 25043054 PMC4249766

[B46] Zhang M. Sun H. Zhao S. Wang Y. Pu H. Wang Y.. (2017). Expression of PD-L1 and prognosis in breast cancer: a meta-analysis. Oncotarget 8, 31347–31354. 10.18632/oncotarget.15532 28430626 PMC5458212

[B47] Zhang Y. Kang S. Shen J. He J. Jiang L. Wang W.. (2015). Prognostic significance of progracmmed cell death 1 (PD-1) or PD-1 ligand 1 (PD-L1) expression in epithelial-originated cancer: a meta-analysis. Medicine 94:e515. 10.1097/MD.0000000000000515 25674748 PMC4602735

[B48] Zheng P. Zhou Z. (2015). Human cancer immunotherapy with PD-1/PD-L1 blockade. Biomark. Cancer 7, 15–18. 10.4137/BIC.S29325 26448693 PMC4578571

[B49] Zhu H. Qin H. Huang Z. Li S. Zhu X. He J.. (2015). Clinical significance of programmed death ligand-1 (PD-L1) in colorectal serrated adenocarcinoma. Int. J. Clin. Exp. Pathol. 8, 9351–9359. 26464688 PMC4583920

[B50] Zou W. Wolchok J. D. Chen L. (2016). PD-L1 (B7-H1) and PD-1 pathway blockade for cancer therapy: mechanisms, response biomarkers and combinations. Sci. Transl. Med. 8:328rv4. 10.1126/scitranslmed.aad7118 26936508 PMC4859220

